# Interventional heart catheterization to close atrial septal defect, patent ductus arteriosus, ventricular septal defect in a 3.5-year-old girl; a case report study

**DOI:** 10.1093/jscr/rjae161

**Published:** 2024-05-03

**Authors:** Zahra Kamiab, Reza Derakhshan

**Affiliations:** Community Medicine Department, School of Medicine, Rafsanjan University of Medical Sciences, Rafsanjan 7717933777, Iran; Pediatrics Department, School of Medicine, Kerman University of Medical Sciences, Kerman 7616913555, Iran

**Keywords:** congenital heart defects, atrial septal defect, patent ductus arteriosus, ventricular septal defect, pulmonary hypertension, catheter intervention

## Abstract

The aim of this study was to introduce an interventional heart catheterization to close patent ductus arteriosus (PDA), ventricular septal defect (VSD), atrial septal defect (ASD), and pulmonary hypertension without complications from open heart surgery and a 3-day hospitalization period. PDA, VSD, and ASD are among the most common abnormalities associated with various complications. This case is a 3.5-year-old girl with frequent lung infections and Failure to thrive. Treatment in the first stage aims to close the PDA using Amplatzer ADO II type AGA, size 5-6 mm and ASD using Amplatzer Septal Occluder size 15 mm. The patient was discharged the next day. Six months later, a successful interventional closure of the VSD was performed using Lifetech Symmetric Amplatzer membranous size 12 mm and patient was discharged 2 days after. All these defects were corrected without open heart surgery and the need for long-term Intensive care unitsadmission.

## Introduction

Congenital heart defects (CHDs) are among the most common types of congenital defects in neonates that can affect their heart structure and functions. These defects occur when the heart or blood vessels do not develop properly in the uterine [1]. Relevant treatments in these neonates are thus especially important, and with the advancement of medical care and new treatments, these neonates are living longer. The incidence of CHD varies from 4 to 50 per 1000 live births [2], and in 2017, out of ~260 000 CHD-related deaths, 180 000 occurred in neonates [3]. Patent ductus arteriosus (PDA), ventricular septal defect (VSD), and atrial septal defect (ASD) are among neonatal CHDs [1].

PDA is one of the heart disorders of newborns, which accounts for 5–10% of all CHDs and its incidence rate is 1 in 2000 births [4]. This disorder can cause intracerebral hemorrhage, necrotizing enterocolitis, and pulmonary dysplasia in neonates [5]. It also causes congestion, pulmonary edema, and disruption of oxygen exchange through by creating left-to-right shunt and increasing pulmonary artery blood flow [6]. VSD is the most common CHDs, affecting 42 cases out of every 10 000 neonates [1]. Diagnosis, determination of the size, and location of VSD by echocardiography during infancy play an important role in improving the patient prognosis. These patients are at risk of pulmonary hypertension [7]. ASD occurs when there is a failure to close the communication between the right and left atria and includes defects involving both the true septal membrane and other defects that allow communication between both atria [8]. According to Centers for Disease Control and Prevention (CDC), ASD affects 13 neonates out of every 10 000 births [1].

CHDs can be managed and treated using various methods such as catheter intervention or surgery. PDA can be closed at any time. In asymptomatic patients, it is recommended to delay treatment until 6 to 12 months to ensure lower risk. While surgical closure of ASD is effective and has a lower mortality, complications such as sternotomy and thoracotomy are unavoidable. Currently, surgery is mostly performed for ASDs with weak septa, which are difficult to close with percutaneous techniques, or failed transcatheter aortic valve replacement cases. The appropriate treatment in patients with partially closed VSD causing aortic insufficiency is surgery and resuspension of aortic valve, which should be carried out before the age of 6 to 12 months [9]. The purpose of the present study was to report a case of a two-stage interventional heart catheterization to close ASD, PDA, and VSD and control pulmonary hypertension in a 3.5-year-old girl within a 3-day hospitalization.

## Case report

The studied case included a 3.5-year-old girl, weighing 11 kg, born as a singleton, which was referred to a hospital in Kerman due to frequent and recurring lung infections and Failure to thrive. Inappropriate general condition and respiratory distress were observed in the examination. Cardiac examination indicated the presence of a 3/6 systolic murmur in the left sternal border, fine rales in the peripheral part of the lung, and a bounding pulse. The chest X-ray showed presence of cardiomegaly, pulmonary vascular markings, and prominence of the pulmonary segment. Sinus rhythm, normal heart axis, large left and right ventricles were observed in the patient electrocardiography. Echocardiography also indicated the presence of situs solitus, synchronized diaphragmatic stimulation, left ventricular hypertrophy , right ventricular hypertrophy, tubular and F type PDA, secundum ASD with size 15 mm, Premembrance VSD with PG 45 mmHg and size 8.5 mm, tricuspid regurgitation 50 mmHg, and dilated pulmonary artery. Lateral ROA (root angiogram) aortogram showed PDA F type. Also, left anterior oblique injection indicates perimembranous VSD and pulmonary artery pressure 50 mmHg.

**Figure 1:**
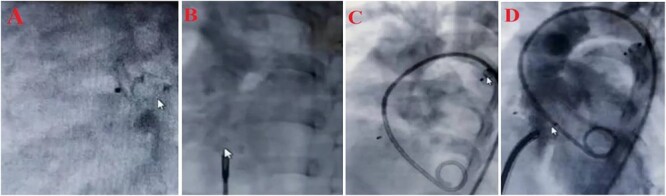
Treatment steps (A) close PDA by Amplatzer ADO II type AGA, size 5–6, (B) close ASD by Amplatzer Septal Oclotec size 15, (C) after closing PDA and ASD, D: close VSD by Amplatzer membranous, lifetec type symetric، size 12, (D) echocardiography four-chamber view after intervention.

Since ASD, PDA, VSD, and pulmonary hypertension were diagnosed; it was decided to perform an interventional heart catheterization. For this purpose, PDA was closed in one step using Amplatzer ADO II type AGA, size 5–6, and then Amplatzer Septal Occluder size 15 was used to close ASD. Finally, the patient was discharged after the first stage treatment process. Six months later, a successful therapeutic interventional closure of the VSD was performed using Lifetech Symmetric Amplatzer membranous size 12 mm. Due to the lack of access to the coil, the smallest and most suitable device was used for the patient. After the treatment process, Echocardiography was used to show the position of ASD amplatzer and VSA amplatzer and there was no residual in VSD and ASD, and complications in the integrity of the aortic valve (without Aortic regurgitation or Aortic stenosis) (Fig. 1). Finally, the patient was discharged with a good general condition and no complications from open heart surgery. The treatment was successfully completed within 3 days of hospitalization. Therefore, the short length of stay in a public hospital led to a significant reduction in patient costs.

## Discussion

CHDs are the most common congenital disorders associated with long-term perinatal complications and mortality. Although ASD, PDA, and VSD are each common disorders and account for ~10% of CHD cases, the combination of these defects, without other significant cardiac lesions, is rare [1, 10]. These cardiac lesions usually lead to a left-to-right intracardiac shunt and ultimately increased pulmonary blood flow. ASDs generally cause right ventricular volume overload, which may lead to right heart failure and atrial arrhythmias. On the other hand, left-to-right shunts at the atrial level or great arteries are associated with volume load in the left ventricle, and cause an increase in left ventricular end-diastolic pressure and pulmonary hypertension through a significant increase in pulmonary venous return, which, in turn, lead to symptoms of congestive heart failure, inability to grow, feeding problems, and diaphoresis [11].

In the present study, an algorithm was presented for diagnosis and simultaneous treatment of ASD, PDA, and VSD as cardiac disorders in a patient with frequent lung infections and confirmed CHD. As mentioned earlier, when the disorders were diagnosed according to the history, examinations, and echocardiography, the decision was made to implement the two-stage treatment process. It should be noted that the open heart surgery was the routine treatment for this patient at this age and low weight. For this purpose, retrograde PDA treatment was performed in the first stage, using Amplatzer ADO II type AGA, 5-6 mm without pulmonary stenosis and coarctation of the aorta. ASD closure was performed using Amplatzer Septal Occluder size 15. In the second stage within 6 months after the first procedure, the VSD was successfully closed using a Lifetech Symmetric Amplatzer membranous size 12 mm. In the study by Aishima et al., a report was presented on the successful treatment of VSD and PDA associated with pulmonary hypertension in an infant with left lung agenesis. Patch was used for VSD closure and PDA was transplanted under cardiopulmonary bypass [12]. In their study, Prada et al. successfully treated 21 babies who had various defects such as an ostium secundum ASD, PDA, and muscular VSD. Researchers suggested closing this type of disorders using Amplatzer as a safe and effective method [13]. Symptoms in most children with OS-ASD are minor and surgery is generally indicated in preschool age. OS-ASD may develop over time and early treatment is necessary in some cases. Children who have the highest risk of progressing to irreversible pulmonary hypertension need early treatment [13, 14].

## Conclusion

The treatment for a patient with several concurrent congenital heart problems should be performed in the form of a cathetric intervention, if possible. This intervention will be beneficial especially for girl patients considering the shorter length of hospital stay and the absence of chest scars. Treatments of similar CHDs can be investigated in future studies and used in depending on the patient condition.
